# Comparison of chemical treatments to ablate the lateral line system in two freshwater fishes, bumblebee goby *Brachygobius doriae* and silver hatchetfish *Gasteropelecus* sp.

**DOI:** 10.17912/micropub.biology.001798

**Published:** 2025-10-02

**Authors:** Hridey Kapoor, Jeremy Levin, Anais Azevedo, Shrija Chhetri, Kyle Lassen, Jia Zheng, Margot A.B. Schwalbe

**Affiliations:** 1 Lake Forest College, Lake Forest, Illinois, United States

## Abstract

Chemically ablating the lateral line system is a useful tool to temporarily and non-invasively inactivate this sensory system, so that the role of this sensory system can be revealed in behaviors relying on flow sensing. Here we determined species-specific lateral line ablation treatments for two freshwater fishes, bumblebee gobies (30 µM gentamycin for 24 hours) and silver hatchetfish (200 µM neomycin for 10 hours in Ca ^2+^ free water), for future behavioral experiments. We recommend that investigators fully vet lateral line ablation treatments prior to behavioral experiments, especially for non-model species.

**Figure 1. 4-di-2-ASP staining verified chemical ablation of canal and superficial neuromasts of the lateral line system in two freshwater fishes, bumblebee goby Brachygobius doriae and silver hatchetfish Gasteropelecus sp.**
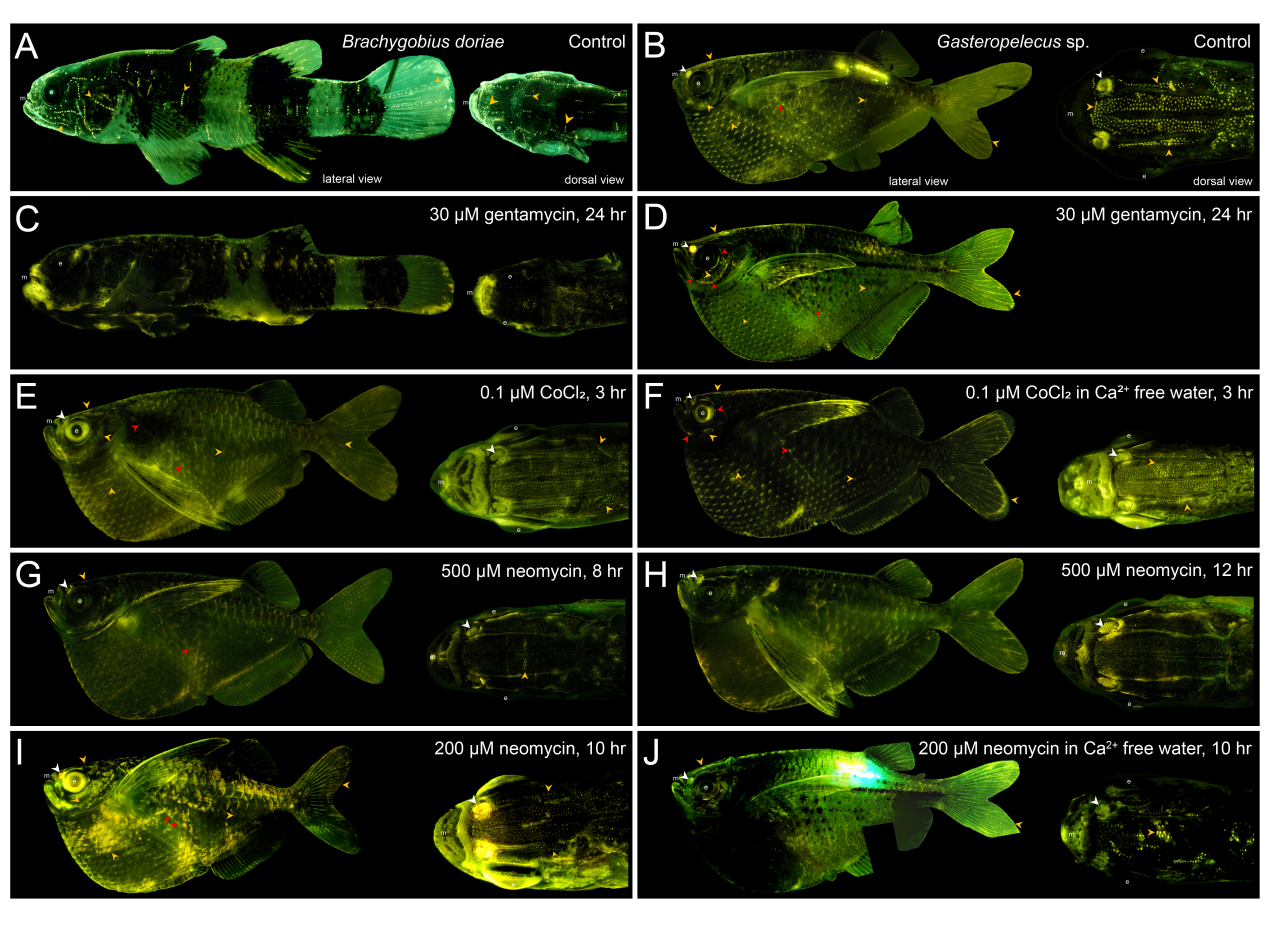
Lateral and dorsal views of a normal, untreated bumblebee goby (A) and a silver hatchetfish (B) show metabolically active neuromasts as bright yellow dots. Representative photomicrographs of a bumblebee goby treated with 30 µM gentamycin for 24 hours (C), and silver hatchetfish treated with 30 µM gentamycin for 24 hours (dorsal view not available) (D), 0.1 µM CoCl _2_ for 3 hours (E), 0.1 µM CoCl _2_ in Ca ^2+^ free water for 3 hours (F), 500 µM neomycin sulfate for 8 hours (G), 500 µM neomycin sulfate for 12 hours (H), 200 µM neomycin sulfate for 10 hours (I), or 200 µM neomycin sulfate in Ca ^2+^ free water for 10 hours (J). Examples of large canal neuromasts are indicated with red arrows and patches of superficial neuromasts are indicated with orange arrows. Olfactory tissue is indicated by white arrows in silver hatchetfish (not visible in views of bumblebee gobies). Note partial fluorescence in neuromasts (>10%) in D-G and I, and fluorescence caused by visible implant elastomer tag in B and J beneath the dorsal fin. *Abbreviations* : e, eye; m, mouth.

## Description

The mechanosensory lateral line system in fish is comprised of hair cell-based neuromasts on the surface of their bodies in stereotypic patterns and it is used to detect surrounding water flows and pressure changes (Mogdans, 2019; Webb, 2023). Neuromasts located on the skin surface are called superficial neuromasts and detect water velocity, while neuromasts located in bony canals on the head or in specialized trunk canal scales along the body are called canal neuromasts and detect water acceleration (Coombs et al., 1989). The role of the lateral line system in various behaviors is often explored in behavioral assays in which experimental fish have an intact or a deactivated lateral line system, so that the outcomes of these experiments can reveal this sensory system’s function. Several studies have physically damaged or chemically ablated the lateral line system to explore this sensory system’s role in many behaviors including, feeding (Schwalbe et al., 2012; Schwalbe & Webb, 2014; Carrillo & McHenry, 2016), escape responses (Mirjany et al., 2011; Stewart et al., 2013), communication (Butler & Maruska, 2015), locomotion (Liao, 2006), rheotaxis (Newton et al., 2023) and schooling (Partridge & Pitcher, 1980; Mekdara et al., 2018, 2021). In addition, this sensory system can serve as an excellent model system to screen for pharmacological and environmental ototoxicity and mechanisms of hair cell death and regeneration (Buck et al., 2012; Monroe et al., 2015; Barrallo-Gimeno & Llorens, 2022; Cirqueira et al., 2024).

The lateral line system can be temporarily disabled by exposing fish to low concentrations of aminoglycoside antibiotics (Song et al., 1995; Harris et al., 2003; Owens et al., 2009; Van Trump et al., 2010) or heavy metal ions (Hernández et al., 2006; Faucher et al., 2008; Butler & Maruska, 2016; Stewart et al., 2017; Hardy et al., 2021). It is good practice to select an appropriate ablation agent, dosage, and exposure time to avoid any unwanted side effects when investigating the role of the lateral line system in various behaviors, and there are tradeoffs with the ablation treatment options. For example, exposure to cobalt chloride will ablate the lateral line system in three hours (Karlsen & Sand, 1987), but over-exposure to cobalt chloride can lead to negative, whole-body side effects (Janssen, 2000) and the alteration of non-target sensory structures, especially olfactory tissue (Butler et al., 2016). Exposure to aminoglycoside antibiotics is generally safer for fish but requires longer treatment durations (Mekdara et al., 2022). Several ablation treatments and their effectiveness are reported, especially for commonly studied fish species and life stages (Karlsen & Sand, 1987; Harris et al., 2003; Murakami et al., 2003; Coffin et al., 2009; Van Trump et al., 2010; Stengel et al., 2017; Stewart et al., 2017; Mekdara et al., 2022). Hair cells in superficial and canal neuromasts regenerate quickly regardless of the type of chemical ablation treatment, with regeneration occurring within three days for superficial neuromasts and up to seven days for canal neuromasts (Thomas et al., 2015; Schwalbe et al., 2016; Hardy et al., 2021; Mekdara et al., 2022). Normal and regenerating hair cells in neuromasts can be visualized with vital fluorescent stains, including 4-di-2-ASP, DASPEI, and FM1-43 (Van Trump et al., 2010; Nakae et al., 2012), making fluorescent microscopy a useful tool to visualize normal, successfully ablated, or regenerating hair cells in superficial and canal neuromasts.

Here, we tested several ablation treatments using two freshwater teleosts, bumblebee gobies ( *Brachygobius doriae* ) and silver hatchetfish ( *Gasteropelecus* sp.), in preparation for behavior experiments linking the structure and function of their lateral line systems. These fishes have different lateral line system morphologies and present unique flow sensing behaviors. Bumblebee gobies have a reduced lateral line canal pattern with proliferation of superficial neuromasts on the head, trunk, and tail (for description, see Nickles et al., 2020), and this pattern likely aids in the detection and capture of prey (Chhetri et al., 2025). Silver hatchetfish have a narrow lateral line canal pattern that is enhanced by hundreds of superficial neuromasts distributed on their head and body and this arrangement may help detect approaching predators, which triggers them to ballistically jump out of the water (Wiest, 1995; Levin et al., 2025). We selected a lateral line ablation treatment for these two species based on previous studies for its efficacy and safety before we began behavioral experiments. We hypothesized that an ototoxic treatment of 30 µM gentamycin sulfate in tank water for 24 hours would effectively disable the lateral line system in both species and without negative side effects after treatment.

The lateral line systems of normal, untreated bumblebee gobies and silver hatchetfish were easily visualized with strong positive fluorescent staining of hair cells in superficial and canal neuromasts ( Fig. 1A & 1B). All fish treated with lateral line ablation agents survived at least seven days post treatment and showed no signs of stress in their recovery tanks, unless otherwise noted. Neuromasts on treated bumblebee gobies were identified on the head and along the trunk and tail in the same location as those on untreated fish, and hair cells within these neuromasts lacked positive fluorescent staining ( Fig. 1C ) and were identified as nonfunctional, and thus chemically ablated. This ablation treatment in silver hatchetfish did not yield the same results. Silver hatchetfish exhibited partial staining (defined as >10% of hair cells fluorescing in all neuromasts) and the decrease in fluorescence was inconsistent in neuromasts located in various regions on the fish’s body ( Fig. 1D ). Further, extending the exposure to 48 and 72 hours (same concentration and solutions changed daily) did not lead to the complete loss of fluorescent staining in neuromasts. Thus, we concluded that these gentamycin treatments did not chemically ablate the lateral line system of silver hatchetfish.

We tested other lateral line system ablation protocols on silver hatchetfish to identify an alternative ablation treatment for this species, which involved cobalt chloride (CoCl _2_ ) (Schwalbe et al., 2012; Mekdara et al., 2022) and neomycin sulfate (Harris et al., 2003; Coffin et al., 2009). Silver hatchetfish exposed to 0.1 µM CoCl _2_ in either tank water or calcium-free water (DI water with salt composition following Karlsen & Sand, 1987) for 3 hours exhibited partial fluorescent staining in hair cells compared to untreated individuals ( Fig. 1E & 1F), and thus was not fully ablated. Further, silver hatchetfish treated with 500 µM of neomycin sulfate for 8 hours had reduced fluorescent staining compared to untreated individuals ( Fig. 1G ). Extending the treatment to 12 hours at this concentration resulted in a complete loss of fluorescent staining in hair cells ( Fig. 1H ) but fish did not survive beyond 12 hours post-treatment. Silver hatchetfish treated with 200 µM neomycin sulfate for 10 hours yielded near complete loss of positive staining ( Fig. 1I ) and healthy post-treated fish, and 200 µM neomycin sulfate for 10 hours in calcium-free water led to the complete loss of positive fluorescence in hair cells (=chemically ablated; Fig. 1J ) and healthy fish seven days post-treatment. Thus, we selected this treatment, 200 µM neomycin sulfate for 10 hours in calcium-free water, as the ablation treatment prior to behavioral experiments.

This study affirms the importance of systematically evaluating lateral line system ablation protocols prior to behavioral experiments, especially with non-model fish species. Our results demonstrate that common lateral line ablation treatments do not result in the same desired outcome in all fish species. Aminoglycoside antibiotics and heavy metal ions are effective at ablating the lateral line system because they interfere with hair cell function and cause hair cell death at certain concentrations and exposure durations (Becker, 2013; Monroe et al., 2015). Calcium ions can alter their ablation efficacy, as high levels of calcium can protect hair cells from these ablation agents (Karlsen & Sand, 1987; Coffin et al., 2009). In addition, hair cells in neuromasts regenerate within days once removed from an ablation treatment (Pinto-Teixeira et al., 2015; Schwalbe et al., 2016; Hardy et al., 2021; Mekdara et al., 2022). Thus, it is important for investigators to confirm the ablation treatment and the time course of hair cell regeneration in their fish species with additional fluorescent staining, so they are confident that their fish have a fully deactivated lateral line system during behavioral experiments.

Variations in lateral line morphology found in bumblebee gobies, silver hatchetfish, and other bony fishes more generally likely influence the success of certain chemical ablation treatments. For example, the number of canal and superficial neuromasts on a fish’s body, as well as their size and shape, cupula properties, and the number of hair cells within a neuromast, may influence the efficacy of certain ablation treatments. Hair cell properties may also contribute to ablation treatment effectiveness, and it is not clear if or how hair cell properties vary across closely and distantly related fish species. Connecting why some chemical ablation treatments are more effective than others at disabling lateral line systems in certain fish warrants further study.

Monitoring the fish after the ablation treatments and fluorescent microscopy was important for future behavioral experiments. The gentamycin and neomycin treatments were safe for their respective species, as fish did not appear stressed and they ate and swam normally in their recovery tanks for at least seven days post-treatment. A mismatch in ablation methodology can lead to confounding variables in behavioral experiments, including incomplete hair cell ablations, loss of other sensory modalities, toxicity, and reduced survivorship (Janssen, 2000; Butler et al., 2016), as well as misidentifying regenerating neuromasts (Song et al., 1995; Van Trump et al., 2010). Thus, confirming that the ablation treatment did not compromise the health or survival of the treated fish gave us confidence that any differences in the behavior of normal versus treated fish were due to the loss of the lateral line system and not other factors.

## Methods

**Animals**

We obtained bumblebee gobies and silver hatchetfish from a local commercial supplier (Fish Planet, Deerfield, IL). Fish were housed by species in conditioned tap water (="tank water," pH adjusted and treated for chloramines) and in small groups (≤5/tank, 38 or 151 L) following standard laboratory conditions under 12:12 h light/dark cycles and were fed daily. Some silver hatchetfish were implanted with a visible implant elastomer tag (Northwest Marine Technology, Inc., Anacortes, WA) for identification (visible in Fig. 1B & 1J). All experiments in this study were conducted in accordance with the guidelines and approved protocols for animal care and use at Lake Forest College (protocol #2023-001).

**Lateral Line Ablation Treatments**

We tested established lateral line ablation protocols on two different fish species in preparation for behavioral experiments. Individual fish experienced only one ablation treatment and were randomly assigned to their treatment. Bumblebee gobies (n=5, TL=23-29 mm) were untreated or treated with 30 µM gentamycin (10 L) for 24 hours. Silver hatchetfish (n=10, TL=40-50 mm) were untreated or treated with one of several ablation protocols (10 L), as established dosages and treatment durations were either ineffective or lethal to this species. We tested some ablation agents in calcium-free water (Karlsen & Sand, 1987), as the lack of calcium in treatment water can alter the calcium channels in neuromast hair cells and ensure the chemical ablation (Coffin et al., 2009). DI water for the calcium-free water was generated by a Barracuda RO/DI System (AquaFX, Winter Park, FL) that filtered municipal tap water, and filtered water was regularly monitored for several parameters, including presence of calcium.

**Lateral Line System Visualization**

We assessed the effectiveness of the lateral line system ablation treatments by comparing fluorescence staining results from normal, untreated and treated fish. Vital fluorescent stains, such as 4-di-2-ASP, DASPEI, and FM1-43, enter mitochondrially active cells and are a useful tool to visualize active hair cells in neuromasts. Thus, we assumed the lack of any positive staining of hair cells in a treated fish was due to the inactivation of hair cells in the neuromasts (Van Trump et al., 2010; Schwalbe et al., 2016) and the fish’s lateral line system was chemically ablated. Partial (>10%) or normal fluorescence of neuromast hair cells in a treated fish indicated that some or all hair cells remain functional and thus the lateral line system is not chemically ablated.

Individual fish were stained with 63 µM 4-di-2-ASP in conditioned tap water (≥250 mL, tap water treated with API StressCoat) for five minutes. For imaging, fish were anesthetized with buffered tricaine (0.01%) and were positioned on a Sylgard-lined petri dish filled with 0.01% buffered tricane. Some fish were euthanized with an overdose of buffered tricaine (0.02%) for high-quality images. Hair cells in superficial and canal neuromasts were imaged under a stereo microscope (Leica M165F) with a green fluorescent protein blue emission filter (wavelength = 425/60 nm excitation range). Fish were illuminated with a fluorescent lamp (NightSea, SFA-RB, 440-460 nm excitation) and all images were taken with a digital camera (Accu-Scope, Excelis 4K) mounted on the microscope. For select fish, images were stitched and blended together (using Adobe Photoshop CS, V. 23.5.1, Ottawa, Canada) for a complete (or mostly complete) view of the fish. Anesthetized fish were quickly revived by placing them in fresh tank water and were monitored in a recovery tank (19-38 L) for at least seven days following this procedure.

## Reagents

Table 1: fluorescent dye, anesthetic, and lateral line ablation agents used in this study

**Table:** 

**Chemical**	**Source**	**Catalog #**	**CAS**
4-(4-Diethylaminostyryl)-1-methylpyridinium iodide (4-di-2-ASP)	Avantor	102989-614	105802-46-8
Tricaine methanesulfonate (MS-222)	Avantor	200000-440	886-86-2
Cobalt Chloride Hexahydrate	Avantor	89230-012	7791-13-1
Gentamycin Sulfate	Avantor	97061-370	1405-41-0
Neomycin Sulfate Hydrate	Thermo Scientific Chemicals	J61499-14	1405-10-3
